# A salivary secretory protein from *Riptortus pedestris* facilitates pest infestation and soybean staygreen syndrome

**DOI:** 10.1111/mpp.13323

**Published:** 2023-03-14

**Authors:** Shiqi Shan, Yue Huang, Chunyun Guo, Biao Hu, Hehong Zhang, Yanjun Li, Jianping Chen, Zhongyan Wei, Zongtao Sun

**Affiliations:** ^1^ State Key Laboratory for Managing Biotic and Chemical Threats to the Quality and Safety of Agro‐products, Key Laboratory of Biotechnology in Plant Protection of MARA and Zhejiang Province Institute of Plant Virology, Ningbo University Ningbo China

**Keywords:** cell death, defence response, *Riptortus pedestris*, salivary protein, soybean staygreen syndrome

## Abstract

The bean bug (*Riptortus pedestris*), one of the most important pests of soybean, causes staygreen syndrome, delaying plant maturation and affecting pod development, resulting in severe crop yield loss. However, little is known about the underlying mechanism of this pest. In this study, we found that a salivary secretory protein, Rp614, induced cell death in nonhost *Nicotiana benthamiana* leaves. NbSGT1 and NbNDR1 are involved in Rp614‐induced cell death. Tissue specificity analysis showed that Rp614 is mainly present in salivary glands and is highly induced during pest feeding. RNA interference experiments showed that staygreen syndrome caused by *R. pedestris* was significantly attenuated when *Rp614* was silenced. Together, our results indicate that Rp614 plays an essential role in *R. pedestris* infestation and provide a promising RNA interference target for pest control.

## Graphical abstract

This study identified a salivary effector (Rp614) of *Riptortus pedestris* that is involved in causing soybean staygreen symptoms by suppressing soybean defence.

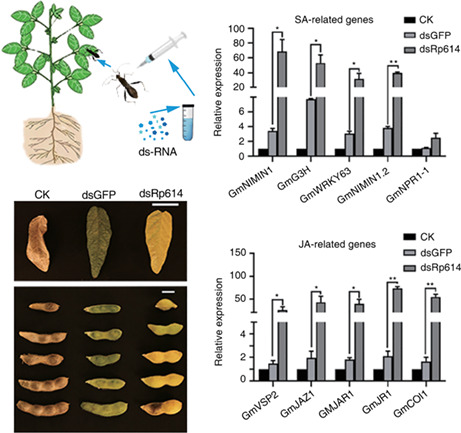

## 1 INTRODUCTION

Soybean (*Glycine max*), one of the most important oil crops, is widely used as food and forage globally (Liu et al., 2008). However, pest insects that feed on pods and seeds, especially *Riptortus pedestris*, cause enormous economic losses to the soybean industry (Rahman & Lim, 2017). *R. pedestris* is a polyphagous pest that is widely distributed in soybean‐growing areas (Jung & Lee, 2019; Li et al., 2021). Our recent study has shown that *R. pedestris* feeding causes soybean staygreen syndrome, as shown by delayed leaf and stem senescence, abnormal pods, and aborted seeds (Wei et al., 2023). During feeding, *R. pedestris* inserts its sucking mouth into the tissues of the host plant to acquire water and nutrients, resulting in a reduction in soybean yield and seed quality (Bae et al., 2014; Fu et al., 2021). In recent years, soybean staygreen syndrome has expanded rapidly in soybean‐growing areas in China (Zhang et al., 2022). However, the mechanisms by which *R. pedestris* causes soybean staygreen syndrome remain obscure.

In nature, plants and herbivorous insects have been engaged in a co‐evolutionary arms race (Erb et al., 2012; Stahl et al., 2018). Hemipterans, including *R. pedestris*, are major pests on crops, causing plant damage by piercing crop plants with their needle‐like mouthparts. During feeding, these insects inject secreted saliva into plant tissues to fix and digest nutrients, acting as effectors to facilitate feeding on the host plant (Fu et al., 2021; Huang et al., 2021). In recent years, with continuous technological advances, some salivary proteins acting as effectors were identified from white‐backed planthopper (*Sogatella furcifera*), brown planthopper (*Nilaparvata lugens*), green peach aphid (*Myzus persicae*), potato aphid (*Macrosiphum euphorbiae*), mirid bug (*Apolygus lucorum*), and white flies (Aleyrodidae) (Chaudhary et al., 2014; De Vos & Jander, 2009; Dong et al., 2020; Miao et al., 2018; Rao et al., 2019; Shangguan et al., 2018; Xu, Qian, et al., 2019). Additionally, it is widely recognized that salivary proteins released by insects into plants are transported inside the host, employing a versatile strategy to suppress plant defence responses to establish successful feeding (Ji et al., 2021). For example, expression of the *M. persicae* salivary effector protein Mp55 in leaves of *Nicotiana benthamiana* supports aphid reproduction on the plant (Elzinga et al., 2014). The secreted salivary protein effector Bsp9 of *Bemisia tabaci* manipulates plant resistance by inhibiting the activation of immunity‐related genes regulated by WRKY33, thereby promoting whitefly preference and performance and increasing virus transmission (Wang et al., 2019). Macrophage migration inhibitory factor (MIF), secreted by aphids, inhibits major plant immune responses in leaf tissues, allowing aphids to exploit their host plants (Naessens et al., 2015). Overexpression of the whitefly salivary gland protein Bt56 in plants increases whitefly performance on host plants and promotes whitefly feeding (Xu, Qian, et al., 2019). Recently, the salivary proteins of *R. pedestris* were characterized by transcriptomic and proteomic studies (Dong et al., 2022; Huang et al., 2021). Although these studies have shown that several salivary proteins are associated with the immune response in the nonhost plant *N. benthamiana*, the function of salivary proteins in the *R. pedestris–*soybean interaction remains unclear.

To reveal the role of salivary proteins in *R. pedestris* infestation, we have identified a salivary protein, Rp614, that can cause cell death in *N. benthamiana* leaves. Rp614 self‐interacts and has a cytoplasmic localization. Mutant analysis showed that the full‐length protein is required for Rp614‐induced cell death. Further investigation revealed that the immune components SGT1 and NDR1 are involved in Rp614‐triggered cell death. Expression analysis indicated that *Rp614* was highly expressed in salivary glands and induced during pest infestation. RNA interference (RNAi) assays showed that the expression of soybean defence‐related genes was significantly up‐regulated and staygreen symptoms were markedly alleviated when *Rp614* was silenced in *R. pedestris*. The salivary protein Rp614 is closely associated with foraging and possibly involved in soybean staygreen syndrome caused by *R. pedestris*.

## 2 RESULTS

### 2.1 Salivary protein Rp614 of *R. pedestris* induces cell death in *N. benthamiana*

**FIGURE 1:**
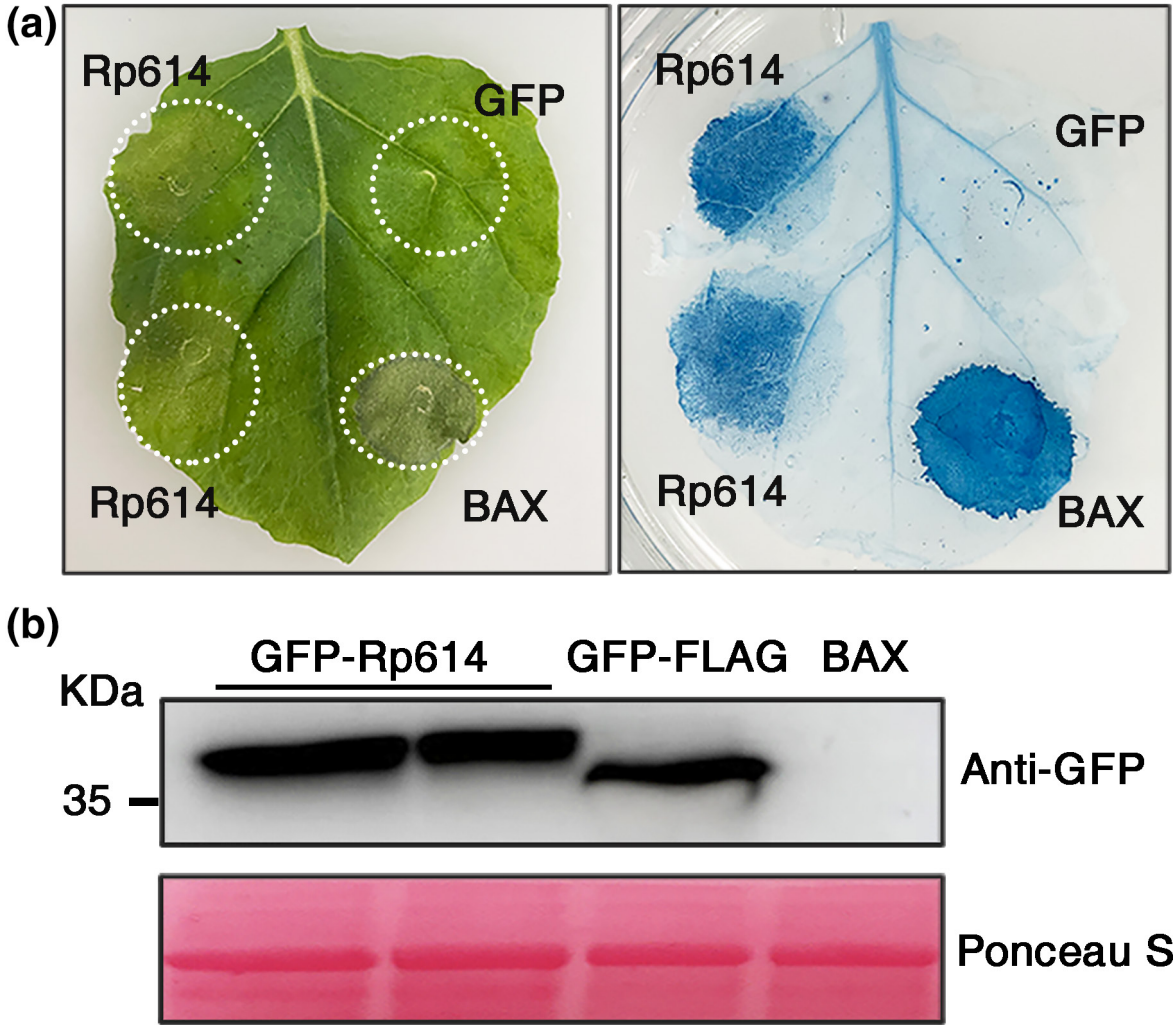
Transient overexpression of *Rp614* in *Nicotiana benthamiana* induces cell necrosis. (a) Leaves of *N. benthamiana* were infiltrated with recombinant vectors GFP‐FLAG (negative control), GFP‐Rp614, and BAX (positive control). Right panel, photographs directly taken at 2 days postinfiltration (dpi). Left panel, photographs taken at 2 dpi after staining with trypan blue. Three independent experiments were performed, with five plants in each experiment. (b) The indicated proteins transiently expressed in *N. benthamiana* were analysed by western blot. Protein loading is indicated by Ponceau S staining.

Many phytopathogen effectors can induce nonhost hypersensitive cell death in *N. benthamiana* (Xu et al., 2021). Our recent research has identified a number of secreted proteins in the salivary glands of *R. pedestris* by transcriptomic and proteomic approaches (Huang et al., 2021). To identify whether these salivary proteins have the ability to induce cell death in *N. benthamiana*, we first cloned the genes encoding these secreted proteins and transiently expressed them in *N. benthamiana* leaves via *Agrobacterium* infiltration. We found that the salivary protein Rp614 induced cell death in leaves. As shown in Figure 1a (left panel), GFP‐FLAG (negative control) caused no obvious symptoms when expressed in *N. benthamiana* leaves, while expression of Rp614 caused obvious cell death in *N. benthamiana* leaves, similar to the positive control BAX (an apoptosis‐promoting protein in the BCL‐2 family). Trypan blue staining, which indicates dead cells and lesions (Figure 1a, right panel), and western blot analysis (Figure 1b) further confirmed that Rp614 was expressed and caused cell death in *N. benthamiana* leaves.

### 2.2 Subcellular localization of Rp614 in *N. benthamiana*

**FIGURE 2:**
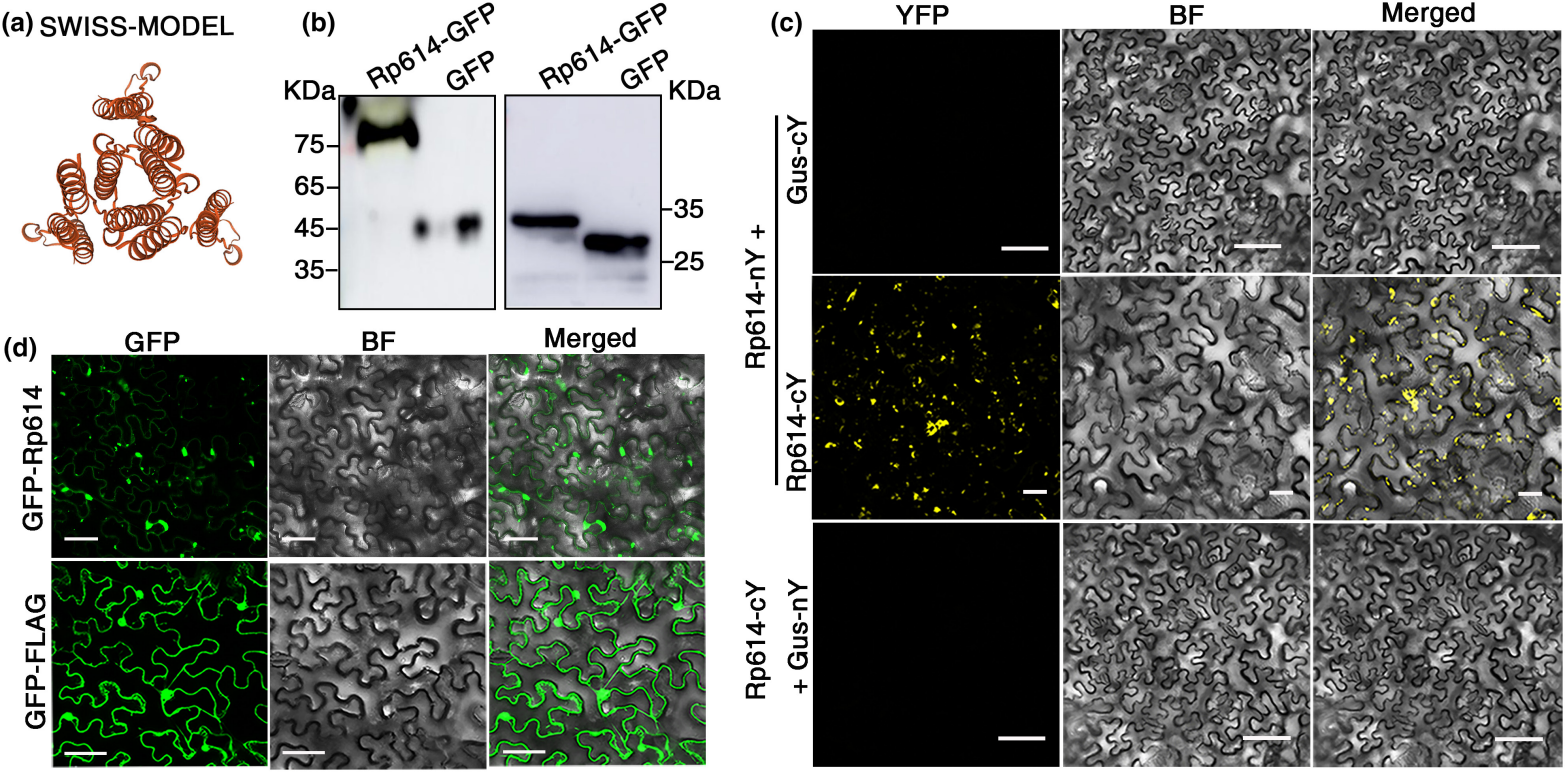
Subcellular localization analysis of the salivary protein Rp614 in *Nicotiana benthamiana* leaf cells. (a) Predicted protein structure of Rp614. (b) The expression of GFP‐Rp614 was analysed by western blot in nondenatured (left panel) and denatured conditions (right panel). (c) Bimolecular fluorescence complementation assays of the interaction between Rp614‐nY and Rp614‐cY. Rp614‐nY + GUS‐cY and Rp614‐cY + GUS‐nY served as negative controls. (d) Subcellular localization of Rp614. Overexpression of GFP‐FLAG (negative control) and GFP‐Rp614 in *N. benthamiana* leaves by agroinfiltration. Confocal images were taken at 24 h postinfiltration. BF, bright field; GFP, green fluorescent protein; Gus, β‐glucuronidase; YFP, yellow fluorescent protein. Scale bars, 20 μm.

Protein sequence analysis revealed that Rp614 is 104 amino acids in length, including a secreted signal peptide (amino acid positions 1–19). The protein has no known function, and no homologous proteins are present in the NCBI database. We tried to predict the protein structure by SWISS‐MODEL (expasy.org). The results showed that Rp614 could form a hexamer (Figure 2a), indicating that Rp614 might self‐interact. To test whether Rp614 interacts with itself, we employed the bimolecular fluorescence complementation (BiFC) method and found that Rp614‐nYFP and Rp614‐cYFP can reconstitute yellow fluorescence and formed concentrated aggregates in *N. benthamiana* cells, but Rp614‐nYFP/GUS‐cYFP or Rp614‐cYFP/GUS‐nYFP did not result in fluorescence in cells (Figure 2c). Additionally, immunoblotting analysis under nondenaturing conditions confirmed that Rp614 formed multimers in *N. benthamiana* leaf cells (Figure 2b). These results suggest that Rp614 can interact with itself and form multimers.

To gain insight into the subcellular localization of Rp614 in the plant cell, we transiently expressed N‐terminal GFP‐tagged Rp614 (without the signal peptide) in *N. benthamiana* leaves and used confocal microscopy to evaluate its localization. As shown in Figure 2d, in GFP‐Rp614‐expressing *N. benthamiana* leaves, green fluorescence was mainly found in the cytoplasm, whereas the control GFP‐FLAG was localized in the nucleus and cytoplasm. These results indicate that Rp614 is localized in the cytoplasm.

**FIGURE 3:**
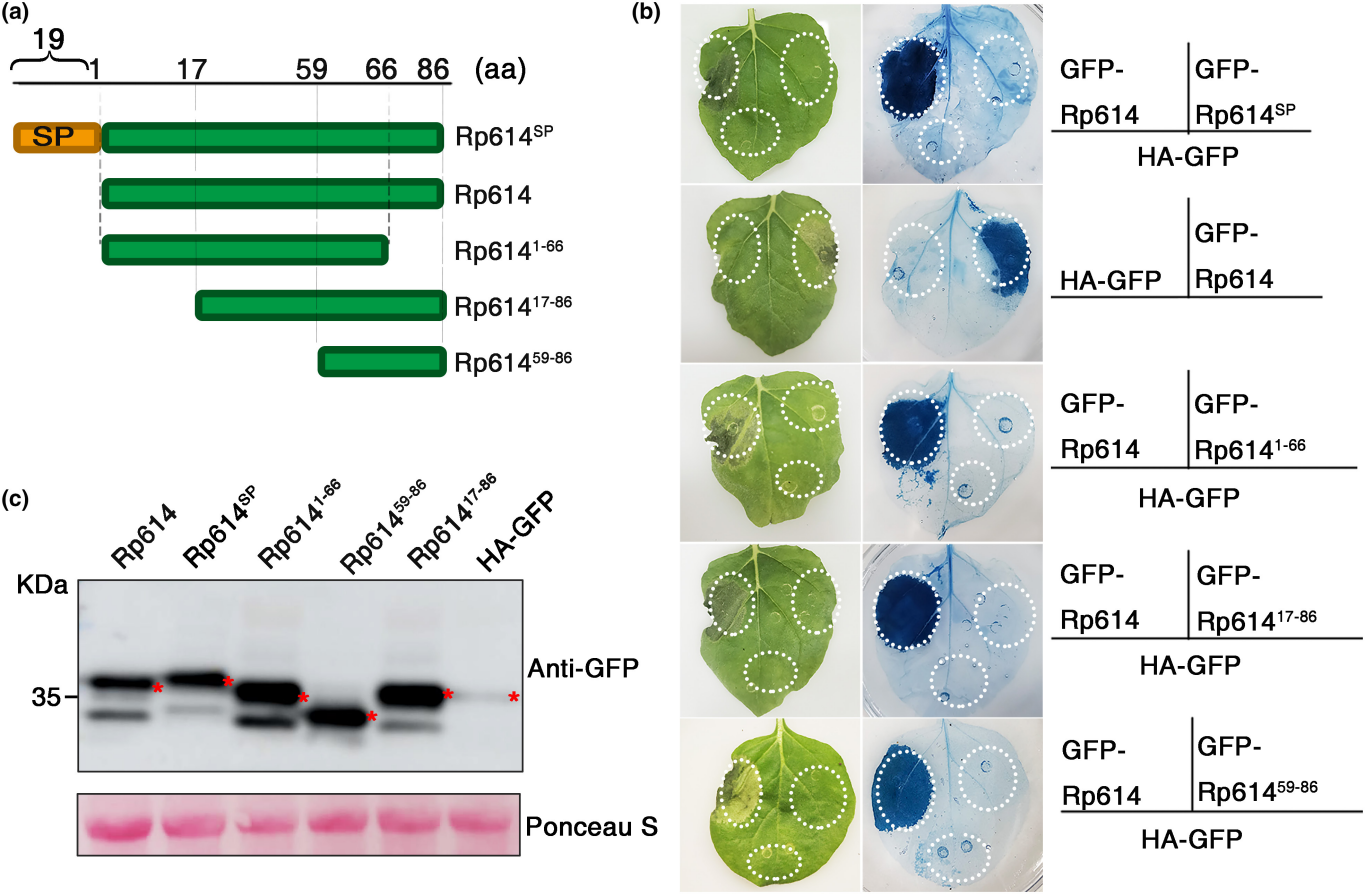
Analysis of cell death triggered by Rp614. (a) Truncated Rp614 mutants examined for cell death‐inducing activity. (b) Representative *Nicotiana benthamiana* leaves infiltrated with *Agrobacterium* strains carrying the indicated genes in vector pBin‐GFP. Trypan blue staining was performed at 2 days postinfiltration (dpi). (c) Rp614 and truncated mutant proteins transiently expressed in *N. benthamiana* were analysed by western blot. Protein loading is indicated by Ponceau S staining. The red asterisk (*) indicates the predicted protein sizes.

Given that Rp614 causes cell death in *N. benthamiana* leaves, we investigated which domain of Rp614 is responsible for this function. We generated several truncated Rp614 mutants and examined their ability to induce cell death (Figure 3a). As depicted in Figure 3b, the areas including amino acid residues 1–66, 17–86, and 59–86 did not induce cell death in *N. benthamiana* leaves. Only Rp614 without the signal peptide triggered cell death in *N. benthamiana* cells. Western blot results revealed that all truncated Rp614 mutants resulted in bands with the predicted molecular weight (Figure 3c). These findings suggest that full‐length Rp614 (without the signal peptide) is necessary for its ability to induce cell death in *N. benthamiana* leaves.

### 2.3 NDR1 and SGT1 are essential for Rp614‐induced cell death in *N. benthamiana*

**FIGURE 4:**
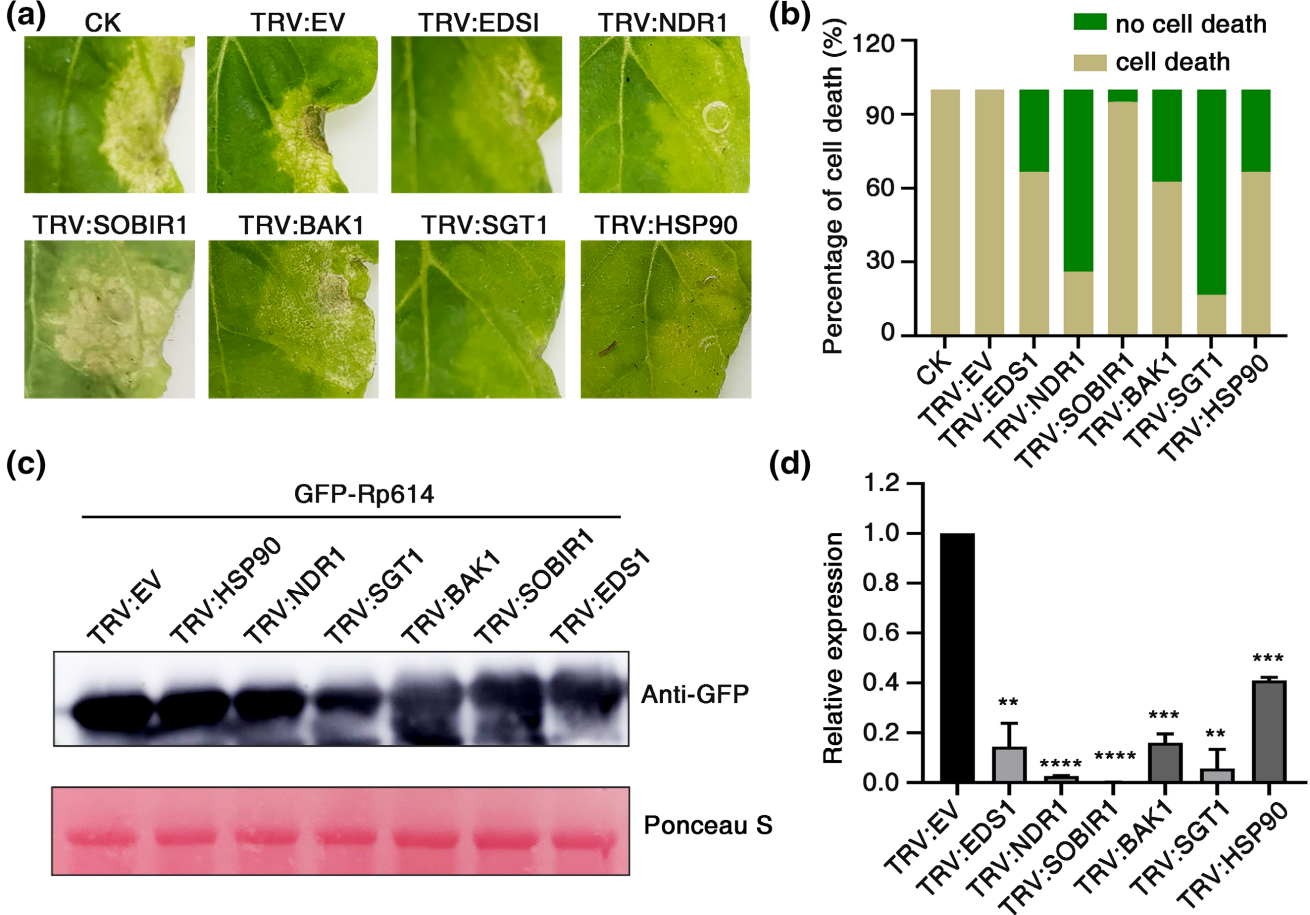
Cell death triggered by Rp614 requires SGT1 and NDR1 in *Nicotiana benthamiana*. (a) Rp614‐triggered cell death in TRV‐silenced *N. benthamiana* leaves. Representative images were taken at 3 days postinfiltration (dpi). Rp614 was transiently expressed in the upper leaves of silenced plants infiltrated with TRV constructs at 16–20 dpi. (b) Quantification of cell death in virus‐induced gene silencing (VIGS)‐treated *N. benthamiana* leaves. *n* ≥ 18. (c) GFP‐Rp614 protein accumulation in silenced leaves as determined by western blot using anti‐GFP. Protein loading is indicated by Ponceau S staining. (d) Relative expression levels of *HSP90*, *NDR1*, *SGT1*, *EDSI*, *BAK1*, *SOBIR1*, and *EDS1* in VIGS‐treated plants as determined by reverse transcription‐quantitative PCR. *Nbactin* was used as the reference gene, and the gene expression levels were normalized to those in TRV:EV (empty vector)‐treated plants. Values are presented as the means ± standard deviation of three biologically independent samples. CK, control. **p < 0.01, ***p < 0.001, ****p < 0.0001, Student's *t* test.

Cell death induced by pathogen effectors is believed to be recognized by the plant pathogen‐associated molecular pattern‐triggered immunity or effector‐triggered immunity systems (Lee et al., 2018; Schulze et al., 2022; Yuan et al., 2021). The receptors and signal transduction pathways involved include the receptor‐like kinase SOBIR1, BAK1 (Albert et al., 2015), the genes associated with the activation of resistance (R) proteins NDR1 and EDS1 (Knepper et al., 2011; Knepper et al., 2014; Wiermer et al., 2005), and the genes responsible for the function of R proteins SGT1 and HSP90 (Kanzaki et al., 2003; Lee et al., 2018; Liu et al., 2004). To determine which signalling pathway is involved in Rp614‐induced cell death, tobacco rattle virus (TRV)‐mediated gene silencing (VIGS) was used to silence these genes in *N. benthamiana*. Two weeks after inoculation with *Agrobacterium* carrying the VIGS constructs, we transiently expressed GFP‐Rp614 in these silenced plants, and cell death was scored 3 days later. The results demonstrated that silencing of *SGT1* and *NDR1* significantly compromised Rp614‐induced cell death (Figure 4a,b). The proportion of dead cells in EDS1‐, BAK1‐, or HSP90‐silenced plants was reduced by about 30% compared to that in TRV:empty vector (EV) control plants, while silencing of *SOBIR1* had no obvious effect on Rp614‐induced cell death (Figure 4b). Immunoblotting assays verified the stable expression of GFP‐Rp614 in these silenced plants (Figure 4c). Reverse transcription‐quantitative PCR (RT‐qPCR) assays confirmed that the transcript levels of the target genes were significantly reduced in silenced plants compared with those in control plants (Figure 4d). Thus, SGT1 and NDR1 are involved in Rp614‐induced cell death.

### 2.4 Identification and characterization of Rp614 in *R. pedestris*

**FIGURE 5:**
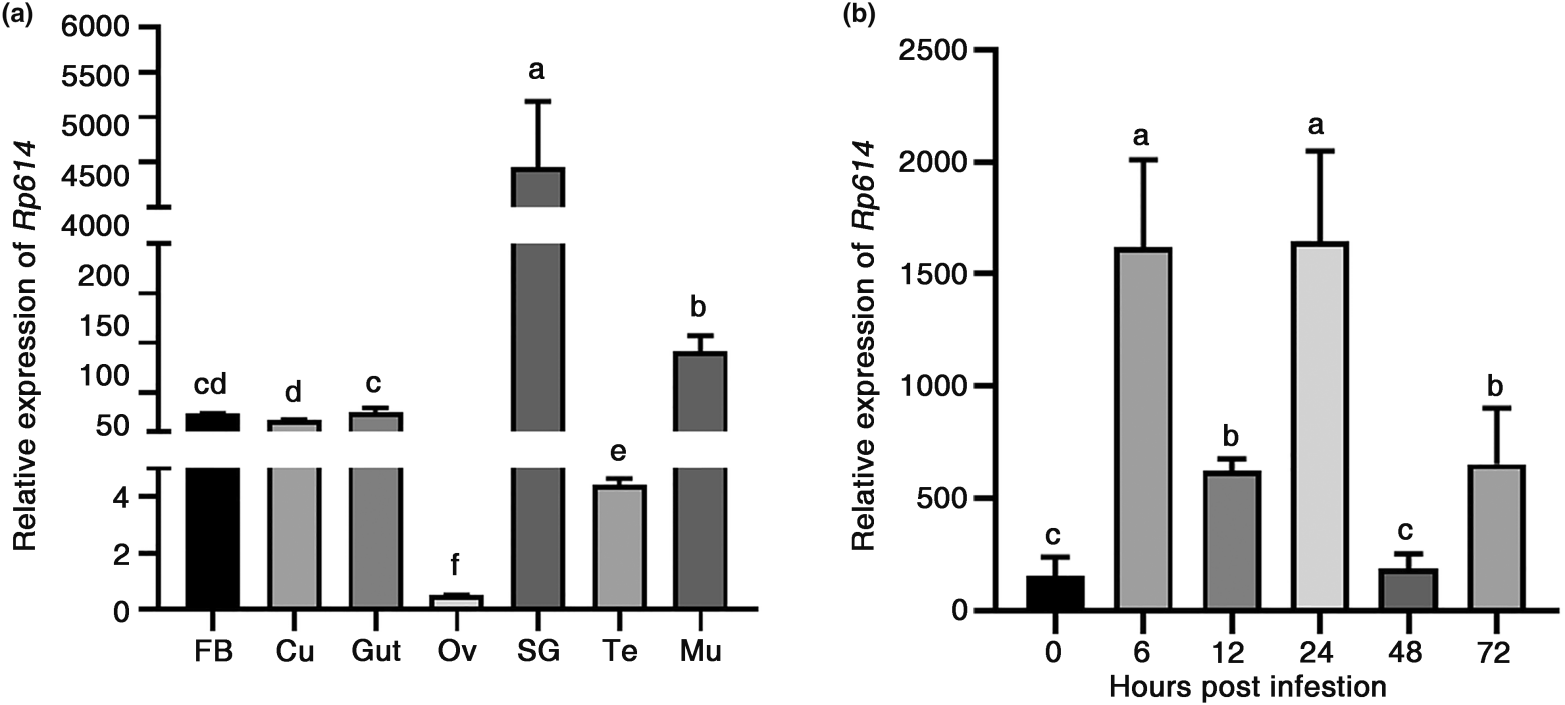
Characterization of Rp614 in *Riptortus pedestris*. (a) Relative expression levels of *Rp614* as determined by reverse transcription‐quantitative PCR (RT‐qPCR) in the following *R. pedestris* tissues: fat body (FB), cuticle (Cu), gut (Gut), ovary (Ov), salivary glands (SG), testis (Te), and muscle (Mu). (b) Relative expression level of *Rp614* after infestation of soybean plants as determined by RT‐qPCR. *RpGAPDH* was used as the reference gene. Values are presented as the means ± standard deviation of three biologically independent samples. Different letters above the bars indicate significant differences among groups (one‐way analysis of variance followed by Duncan's multiple range test, *p* < 0.05).

Considering that the salivary protein Rp614 induces cell death in *N. benthamiana* leaves, we wondered about the expression pattern of this effector. Hence, we analysed the relative expression of *Rp614* in various *R. pedestris* tissues, including the fat body, cuticle, gut, ovary, salivary glands, muscle, and testis, by RT‐qPCR. The results showed that the relative expression of *Rp614* was much higher in the salivary glands than in other tissues (Figure 5a). Next, we collected soybean seeds on which *R. pedestris* had fed and conducted mass spectrometry (MS) analysis. The results showed that one peptide matched part of Rp614 (Figure S1). These results indicate that *Rp614* is highly expressed in salivary glands and is secreted into soybean seeds during *R. pedestris* feeding.

To elucidate the transcript profile of *Rp614* during *R. pedestris* feeding, we analysed the transcript levels of *Rp614* at different times during feeding by RT‐qPCR. The results showed that the expression of *Rp614* was strongly activated when feeding from 6 h, and expression remained high during feeding (Figure 5b). These findings suggest that Rp614 may play a crucial role in *R. pedestris* feeding on soybean plants, as its expression is strongly induced during the feeding process.

### 2.5 The effects of Rp614 on soybean immunity and staygreen symptoms caused by *R. pedestris*

**FIGURE 6:**
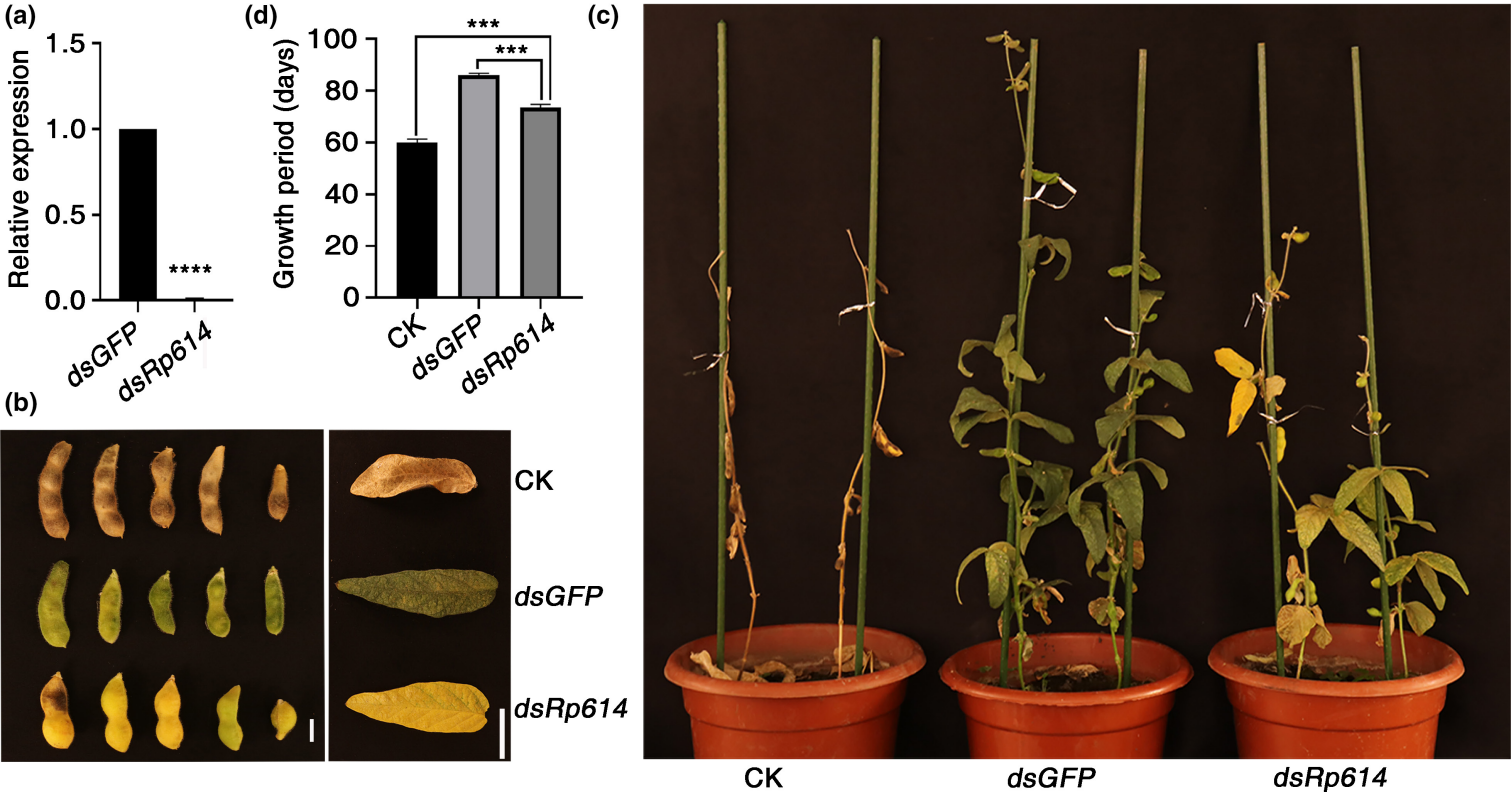
Effects of *Rp614*‐silenced *Riptortus pedestris* infestation on soybean plants. (a) RNAi efficiency of *Rp614* was determined by reverse transcription‐quantitative PCR. *RpGAPDH* was used as an internal reference. (b, c) The appearance of pods (b) and leaves (c) of soybean plants without (CK) or with *dsRp614*‐ or *dsGFP*‐treated *R. pedestris* infestation at the pod stage for 14 days. Representative images were taken 30 days after insects were removed. (d) Growth period of soybean in different treatment groups. Scale bars, 1 cm in (b), 15 cm in (c). Values are presented as the means ± standard deviation of three biologically independent samples, *n* ≥ 6. ***p < 0.001, ****p < 0.0001, Student's *t* test.

**FIGURE 7:**
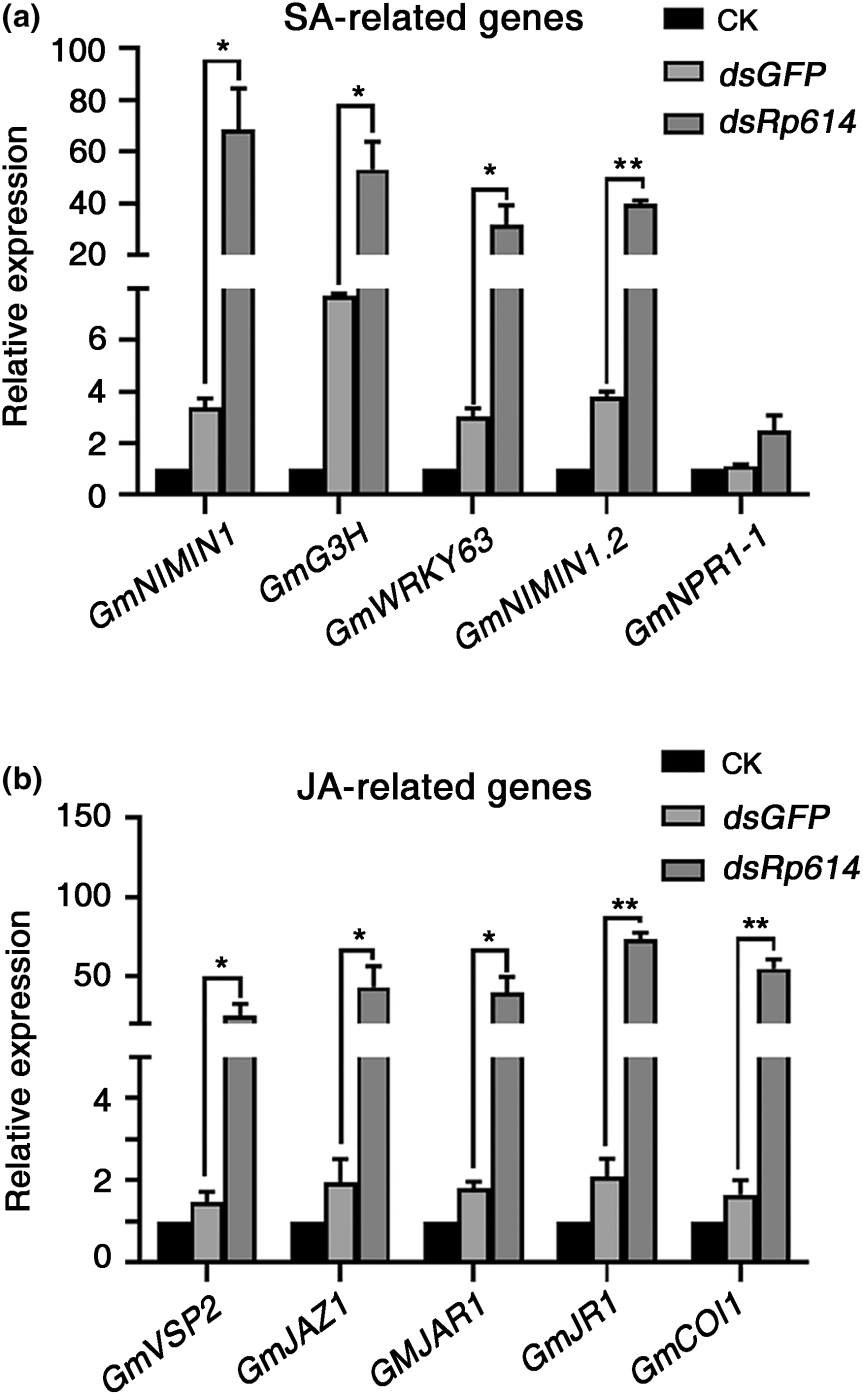
Silencing of *Riptortus pedestris* *Rp614* affects the expression of defence‐related genes. (a, b) Expression levels of salicylic acid synthesis‐related genes (a) and jasmonic acid synthesis‐related genes (b) in soybean leaves after feeding by *dsRp614*‐ or *dsGFP*‐treated *R. pedestris* and in soybean leaves without pest (CK). *GmCYP2* was used as the internal reference gene. Values are presented as the means ± standard deviation of three biologically independent samples. *p < 0.05, **p < 0.01, Student's *t* test.

To investigate the role of Rp614 during *R. pedestris* feeding, we silenced *Rp614* by double‐stranded RNA (dsRNA)‐mediated RNAi. A dsRNA fragment of *Rp614* or *GFP* (negative control) was synthesized and injected into *R. pedestris*. The silencing efficiency of *Rp614* was determined at 14 days postinoculation (dpi). As shown in Figure 6a, the expression of *Rp614* was significantly reduced in *dsRp614*‐treated insects compared to *dsGFP*‐treated insects. These results indicated that *Rp614* was efficiently silenced. As heterologous expression of *Rp614* was associated with plant immunity in *N. benthamiana*, we next investigated the role of *Rp614* in its natural host soybean. Soybean plants at the beginning pod (R3) stage were fed on by *dsRp614‐GFP‐* or *dsGFP*‐treated *R. pedestris*. The expression of salicylic acid (SA)‐ (*GmNIMIN1*, *GmG3H*, *GmWRKY63*, *GmNIMIN1.2*, and *GmNPR1‐1*) and jasmonic acid (JA)‐related genes (*GmVSP2*, *GmJAZ1*, *GmJAR1*, *GmJA1*, and *GmCOI1*) was analysed in these soybean plants to evaluate the effect of *Rp614* on soybean defence. The results showed that the expression of most SA‐ or JA‐related genes was obviously increased in soybean plants fed on by *dsGFP*‐treated *R. pedestris* compared with mock plants (Figure 7a,b), indicating that the SA and JA pathways might be involved in the defence against *R. pedestris* infestation in soybean. However, the expression of these SA‐ and JA‐related genes was significantly up‐regulated in soybean fed on by *Rp614*‐silenced insects compared with soybean fed on by *dsGFP*‐treated insects (Figure 7a,b). These results illustrate that *Rp614* might interfere with the soybean immune response by suppressing the expression of hormonal defence genes during *R. pedestris* infestation.

Because *R. pedestris* causes staygreen syndrome in soybean (Wei et al., 2023; Zhang et al., 2022), we wondered whether *Rp614* plays a role in *R. pedestris* infestation. We carried out *R. pedestris* feeding experiments at the R3 stage of soybean. The soybean plants infested with *dsGFP*‐treated *R. pedestris* developed a typical staygreen syndrome, including delayed leaf and pod senescence and significant yield loss, compared with the mock‐treated plants, which reached maturity normally (Figure 6b,c). These results were consistent with previous results that *R. pedestris* feeding causes staygreen syndrome in soybean (Wei et al., 2023). However, when fed on by *dsRp614*‐treated *R. pedestris*, the symptoms in soybean were significantly alleviated compared with soybean fed on by *dsGFP*‐treated *R. pedestris*, revealing significantly earlier maturity (73.5 ± 1.7 days vs. 86 ± 0.7 days) (Figure 6d). Together, these results suggest that silencing of *Rp614* attenuated the staygreen symptoms caused by *R. pedestris*, indicating that Rp614 plays an essential role in *R. pedestris* infestation.

## 3 DISCUSSION

Herbivorous insects and their host plants have developed dynamic and complex interactions over the course of their co‐evolution. Insects secrete a number of salivary proteins into plants when they feed on them, and these salivary proteins pass through the host plant and affect the life processes of the host plant (Ji et al., 2021). Several molecular mechanisms for the interaction of insect‐secreted proteins with host plants have been reported (Chaudhary et al., 2014; Cui et al., 2019; Du et al., 2009; Rodriguez et al., 2014; Xu, Qian, et al., 2019). However, studies on the identification and function of salivary proteins of *R. pedestris* are limited.

In the present study, we identified Rp614 as a secreted protein in the salivary glands of *R. pedestris* that induces cell death in tobacco cells (Figure 1a). Further analysis of the Rp614 sequence revealed that the protein's function is unknown, and structural predictions and protein interaction methods showed that it is capable of self‐interaction. In addition, subcellular localization showed that Rp614 is localized to the cytoplasm, and we found that Rp614 with its signal peptide does not induce cell necrosis, indicating Rp614 might play essential roles inside plant cells (Figure 3). We applied VIGS to demonstrate that SGT1 and NDR1 are required for the induction of cell death by Rp614 (Figure 4). SGT1 promotes the accumulation of many R proteins in plants and can positively regulate the disease resistance they confer (Azevedo et al., 2006). NDR1 mediates the plant defence response and is involved in the activation of R proteins (Shapiro & Zhang, 2001). The fact that SGT1 is involved in the *R* gene or pattern recognition receptor‐mediated immune signalling suggests that plant leaf necrosis triggered by Rp614 may be due to activation of the plant defence pathway.

*R. pedestris* obtains nutrients by inserting its mouthparts into soybean stems, leaves, pods, and seeds, resulting in delayed leaf and stem senescence, abnormal pods, and aborted seeds, which is known as staygreen syndrome (Bae et al., 2014; Rahman & Lim, 2017; Wei et al., 2023). However, the role of salivary proteins in *R. pedestris* feeding is unclear, and it is not known whether these proteins are involved in the soybean staygreen syndrome. Here we employed dsRNA silencing to investigate the potential interactions between an *R. pedestris* salivary protein and soybean staygreen syndrome. The use of dsRNA silencing of related genes to study and analyse the function of salivary effectors in Hemiptera has been widely reported (Pitino et al., 2011; Xu, Tang, et al., 2019). In the present study, the role of Rp614 in insect feeding on soybean was further investigated by injecting dsRNA to silence the *Rp614* gene. We found that hormonal defence pathways, including SA‐ and JA‐related genes, were significantly activated in soybean fed on by *Rp614*‐silenced insects compared with *dsGFP*‐treated insects (Figure 7), indicating that Rp614 might suppress the soybean immune response by affecting the expression of hormonal defence genes during *R. pedestris* infestation. Additionally, immunosuppressive effectors inducing necrosis have also been reported in various plants. For example, the RXLR effectors PpE4 and AVh238 enhance plant susceptibility to *Phytophthora parasitica* and induce cell death in *N. benthamiana* (Huang et al., 2019; Yang et al., 2017). The staygreen syndrome was further evaluated after *Rp614* was silenced in *R. pedestris*. The results showed that soybean maturation was significantly earlier after infestation by *dsRp614*‐treated *R. pedestris* compared with the *dsGFP*‐treated control. These results indicate that Rp614 plays an essential role in *R. pedestris* infestation. Future studies will focus on the detailed role of Rp614 in the *R. pedestris–*soybean interaction and the underlying mechanism. Overall, this study provides insights into plant damage by *R. pedestris* and the function of insect‐secreted effectors, which may help in establishing soybean pest prevention and control systems in the future.

## 4 EXPERIMENTAL PROCEDURES

### 4.1 Insects and plant materials

*R. pedestris* in this study was captured in a soybean field in Suzhou, Anhui, China in 2019 (33.7°N, 117.0°E). The insects were reared on soybean plants and dried seeds at 26 ± 1°C and 60 ± 5% relative humidity under a 16:8 h (light:dark) photoperiod. *N. benthamiana* was grown in a growth chamber at 23°C with a 16:8 h photoperiod. The soybean used for the pest infestation experiments was an early‐maturing variety, Mengdou 16, and the seeds were sown in plastic pots (26 cm height, 32 cm diameter); two healthy plants were kept in each pot and plants were grown in a greenhouse (25 ± 2°C, 16:8 h photoperiod).

### 4.2 *Agrobacterium tumefaciens* infiltration assays

The full‐length or truncated cDNA sequences of *Rp614* were amplified from *R. pedestris* salivary glands using the primers listed in Table S1 and then cloned into the plant expression vector pBinGFP. These recombinant expression vectors were electroporated (2.2 kV) into *A. tumefaciens* GV3101. Transformants were incubated for 48 h at 28°C in Luria–Bertani liquid medium with 50 mg/mL kanamycin and 25 mg/mL rifampicin. The bacterial cultures were collected by centrifugation at 5000 × *g* for 3 min and resuspended in induction buffer (10 mM MgCl_2_, 10 mM MES pH 5.6, 0.2 mM acetosyringone) to a final concentration of OD_600_ = 2.0. The *Agrobacterium* culture was kept in the dark at 28°C for a minimum of 2 h. The treated *Agrobacterium* solution was infiltrated into approximately 6‐week‐old *N. benthamiana* leaves.

### 4.3 Trypan blue staining

Trypan blue staining was performed as previously described, but with slight modifications (Gao et al., 2022). The leaves were removed, completely submerged in trypan blue staining solution, and then placed in boiling water for 10–15 min until the leaves became transparent. Staining solution was prepared by mixing 20 mL ethanol, 10 mL lactic acid, 10 mL phenol, and 10 mg trypan blue. Finally, the samples were decoloured using chloral hydrate and photographed.

### 4.4 Western blot analysis

*N. benthamiana* leaves were collected and ground into powder in liquid nitrogen. The total denatured proteins were extracted with SDS lysis buffer (100 mM Tris–HCl pH 6.8, 20% SDS, 2% β‐mercaptoethanol) and separated on 10% SDS‐PAGE gels. For nondenatured conditions, protein was extracted with IP lysis buffer containing 40 mM Tris–HCl (pH 7.5), 100 mM NaCl, 4 mM MgCl_2_, 1 mM EDTA, 1% glycerol, and 0.2% Triton X‐100 and separated with nondenaturing PAGE. The corresponding fusion proteins were then incubated with monoclonal mouse anti‐GFP and then transferred to preactivated PVDF membranes to detect the corresponding fusion proteins. Imaging was performed using ECL substrate and a Bio‐Rad ChemiDoc MP imaging system.

### 4.5 BiFC

For the generation of the BiFC vectors, the full‐length cDNA of *Rp614* was amplified by PCR using the primers listed in Table S1 and then cloned into cYFP and nYFP vectors. The recombinant expression vectors were transformed into *A. tumefaciens* GV3101 and infiltrated into *N. benthamiana* leaves. After 24 h, YFP fluorescence was captured using a confocal laser microscope (Nikon).

### 4.6 RT‐qPCR analysis

Total RNA (1.5 μg from different tissues of *R. pedestris* and leaves of *N. benthamiana* and soybean) was pretreated with gDNA wiper mix to eliminate genomic DNA and then reverse transcribed to cDNA using HiScript III qRT Super Mix (Vazyme). qPCR was performed using the SYBR Green Supermix Kit (Vazyme) on a Roche Light Cycler 480 Real‐Time PCR system (Roche) with the following reaction procedure: denaturation at 95°C for 5 min, followed by cycling at 95°C for 10 s and 60°C for 30 s. The data were further analysed by the 2^−∆∆*C*t^ method. The primers used are listed in Table S1.

### 4.7 VIGS in *N. benthamiana*

*A. tumefaciens* GV3101 carrying different pTRV2 constructs was mixed with pTRV1 in equal proportions to a final OD_600_ of 0.25. pTRV2:EV was used as a negative control as described previously (Huang et al., 2019). The lower leaves of *N. benthamiana* plants at the four‐leaf stage were infiltrated and gene silencing efficiency was determined by RT‐qPCR after 2 weeks. The primers used in this study are listed in Table S1.

### 4.8 RNAi application and *R. pedestris* feeding experiments

The dsRNA was synthesized and purified using the T7 High Yield RNA Transcription Kit (Vazyme) according to the instructions, and *dsGFP* was used as a negative control. Primers are listed in Table S1. The synthesized dsRNA (4 µg/mL) was injected into adult *R. pedestris* (1 μL per insect) and insects were placed in the insect rearing chamber for 24 h. Then, adult *R. pedestris* treated with *dsRp614* or *dsGFP* were inoculated onto soybean plants at the pod stage (five insects per plant). Control plants (mock) were placed in nylon mesh cages without insects. Each experiment was replicated at least three times. The state of the plant was assessed by observing leaf and pod colour and the growth period.

### 4.9 Statistical analysis

Differences were analysed using Student's *t* test; *p* ≤ 0.05 was considered statistically significant. All analyses were performed using GraphPad Prism v. 8.0.1.

## CONFLICT OF INTEREST STATEMENT

The authors declare no conflict of interests.

## Supporting information


**Figure S1** Peptide analysis of Rp614 by mass spectrometry.

**Table S1** List of primers used for this study.

## Data Availability

The data that support the results of this study are included in this article and its supplementary materials. Raw reads generated by transcriptomic sequencing have been submitted to the NCBI Sequence Read Archive at https://www.ncbi.nlm.nih.gov/sra under accession number PRJNA671796.
