# Supplementary material for: A salivary secretory protein from Riptortus pedestris facilitates pest infestation and soybean staygreen syndrome
Source: Mol Plant Pathol. 2023 Mar 14;24(6):560–9. doi: 10.1111/mpp.13323 (PMC10189764; doi:10.1111/mpp.13323)
Supplement: Supplementary file 1 — Figure S1 Peptide analysis of Rp614 by mass spectrometry. [file MPP-24-560-s001.docx]

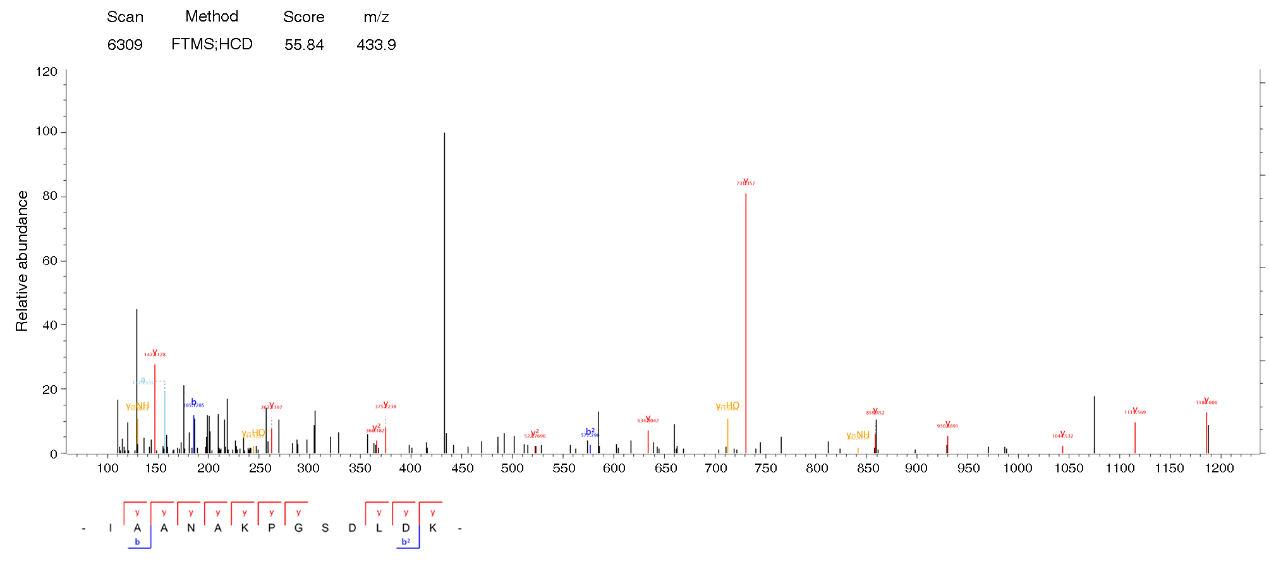


**Figure S1.** Peptide analysis of Rp614 by Mass spectrometry.

The b^+^ ions retain the N terminus of the peptide chain and the charge is retained at the C terminus; the y^+^ ions retain the C terminus of the peptide chain and the charge is retained at the N terminus of the ion.
