# Supplementary material for: A salivary secretory protein from Riptortus pedestris facilitates pest infestation and soybean staygreen syndrome
Source: Mol Plant Pathol. 2023 Mar 14;24(6):560–9. doi: 10.1111/mpp.13323 (PMC10189764; doi:10.1111/mpp.13323)
Supplement: Supplementary file 2 — Table S1 List of primers used for this study. [file MPP-24-560-s002.docx]

**Supplementary Table 1.** Primer sequences were used for this study.

| Usage | Primer name | Primer sequence |
| --- | --- | --- |
| Cloning of sequences | Bin-Rp614-F | ACGAGCTGTACAAGGGTACCATGGGTCCAGCCGATCTTATCAAAG |
|  | Bin-Rp614-R | GCGGACTCTAGTTCATCTAGATTATGCGAATCCTTGGATCGTG |
|  | Bin-Rp614-54-252-InR | GCGGACTCTAGTTCATCTAGAGCGTCCTGCATCAAAGGATG |
|  | Bin-Rp614-SP-InF | ACGAGCTGTACAAGGGTACCATGAAGGTCGTCATTGCATTAC |
|  | Bin-Rp614-231-315-InF | ACGAGCTGTACAAGGGTACCATGGCATCCTTTGATGCA |
|  | Bin-Rp614-105-315-InF | ACGAGCTGTACAAGGGTACCATGGTTGAAAATGGAGTGAACAA |
|  | LIC-Rp614-F | CGACGACAAGACCGTCACCATGGGTCCAGCCGATCTTATCAAAG |
|  | LIC-Rp614-R | GAGGAGAAGAGCCGTCGTTGAGGGGAACGTATCCAGTGG |
| qRT-PCR | qRT-Rp614-F | GGTCCAGCCGATCTTATCAA |
|  | qRT-Rp614-R | GTTTGGCATTCGCTGCTATT |
|  | qGmNIMIN1-F | ATGTTGAACACGGCATTCTC |
|  | qGmNIMIN1 -R | GGTACGGTGTGACTTTCTTG |
|  | qGmG3H-F | GGCGCAGTATATTCCAACTC |
|  | qGmG3H-R | GTTTGGCATGATGGTGTAGG |
|  | qGmNIMIN1.2-F | CCTGACACCAGACCATGATAC |
|  | qGmNIMIN1.2-R | TTATGGTTGTGCGTGGTTG |
|  | qGmWRKY63-F | CACACCTAAAGGCTCATCAC |
|  | qGmWRKY63-R | GTTTGAGACCACCCTGAAAG |
|  | qGmVSP2-F | CATGGCCACAGCTACCAAATT |
|  | qGmVSP2-R | TTGCACGATACCTCCGGAAT |
|  | qGmJAZ1-F | CAAACGGTTCCCCTGAGACA |
|  | qGmJAZ1-R | TGGGTTCCGTTGCGTGAT |
|  | qGmJAR1-F | TACCGACTTGGTGATGTGGTTAAG |
|  | qGmJAR1-R | AGATTGCTACGCCGAACGAA |
|  | qGmJR1-F | AGGCACCCCCGTCATTCTA |
|  | qGmJR1-R | GAGTCCCTTCTTCAACTCCATAGG |
|  | qGmCOI1-F | CTCAGTACTTCGATTGCCTCAAGAG |
|  | qGmCOI1-R | ACGAGCGAGATTCCGAAGATC |
|  | qGmNPR1-1-F | AGGGAGTTTTGAAGTGTGAAGT |
|  | qGmNPR1-1-R | AGGTCCAGGATCAGAGCCAT |
|  | Nbactin -F | ACCATCAATGATCGGAATGG |
|  | Nbactin -R | GCTCATCCTATCAGCAATGC |
|  | NbBAK1q-F | CTGAACGGTTGCTTGTTTATCC |
|  | NbBAK1q-R | TACGCTTCCTTATTGACCACTC |
|  | NbSOBIR1q-F | CTTAGAAAAACTCTCTTTAGC |
|  | NbSOBIR1q-R | TATGGATTGGAGTGACATTATG |
|  | HSP90q-F | GTTTCAGCCTCGACGATCCCA |
|  | HSP90q-R  SGT1q-F1 | GTCAACATCAGCATCCCCAGAA  AGGACACCAGAAGAGCCATG |
|  | SGT1q-R1 | ACCTTCTTTGCACCGACTTCT |
|  | EDS1q-F | GACTTAGGCCTGAGTACAAGAG |
|  | EDS1q-R1 | CTGTATCTTGCTTAATCCTTCCATG |
|  | NDR1q-F | CCTGCCCTTAACAACTCTGAT |
|  | NDR1q-R | GAACACCTTTGTCCTTCATCTTG |
|  | qGmCYP2-F | CATGGCCACAGCTACCAAATT |
|  | qGmCYP2-R | TTGCACGATACCTCCGGAAT |
|  | qRpGAPDH-F | CTGTTGTAGACCTCACTGTT |
|  | qRpGAPDH-R | ATATCCGCACTCATTGTCAT |
| RNAi | dsRp614-F | TAATACGACTCACTATAGGGATGAAGGTCGTCATTGCATTAC |
|  | dsRp614-R | TAATACGACTCACTATAGGGTTGAGGGGAACGTATCCAGTGG |
|  | dsGFP-F | GCGGACTCTAGTTCATCTAGATTAAATTCCCAGACTCAGCC |
|  | dsGFP-R | AATACGACTCACTATAGGGCAGACTACCACACCTAGGACC |
